# A Dynamic Transcriptome Map of Different Tissue Microenvironment Cells Identified During Gastric Cancer Development Using Single-Cell RNA Sequencing

**DOI:** 10.3389/fimmu.2021.728169

**Published:** 2021-10-21

**Authors:** Honghao Yin, Rui Guo, Huanyu Zhang, Songyi Liu, Yuehua Gong, Yuan Yuan

**Affiliations:** ^1^ Tumor Etiology and Screening Department of Cancer Institute and General Surgery, The First Hospital of China Medical University, Shenyang, China; ^2^ Key Laboratory of Cancer Etiology and Prevention in Liaoning Education Department, The First Hospital of China Medical University, Shenyang, China; ^3^ Key Laboratory of GI Cancer Etiology and Prevention in Liaoning Province, The First Hospital of China Medical University, Shenyang, China

**Keywords:** gastric disease, dynamic transcriptome map, tissue microenvironment, single-cell RNA sequencing, biomarkers

## Abstract

Gastric cancer (GC) development trends have identified multiple processes ranging from inflammation to carcinogenesis, however, key pathogenic mechanisms remain unclear. Tissue microenvironment (TME) cells are critical for the progression of malignant tumors. Here, we generated a dynamic transcriptome map of various TME cells during multi-disease stages using single-cell sequencing analysis. We observed a set of key transition markers related to TME cell carcinogenic evolution, and delineated landmark dynamic carcinogenic trajectories of these cells. Of these, macrophages, fibroblasts, and endothelial cells exerted considerable effects toward epithelial cells, suggesting these cells may be key TME factors promoting GC occurrence and development. Our results suggest a phenotypic convergence of different TME cell types toward tumor formation processes in GC. We believe our data would pave the way for early GC detection, diagnosis, and treatment therapies.

## Introduction

Globally, gastric cancer (GC) is the fifth most common cancer tumor (1). GC development undergoes a multi-stage process, from no-atrophic-gastritis (NAG) to chronic atrophic gastritis (CAG) to intestinal metaplasia (IM), and finally GC (2). During this process, gastric mucosa tissue and the tissue microenvironment (TME) undergo dynamic changes (3). The TME includes a variety of cell types (immune cells, fibroblasts, endothelial, etc.) and stromal components (chemokines, cytokines, growth factors, etc.) surrounding epithelial cells. Increasingly, it is recognized that the cellular features of the TME play an important role in enabling tumors to proliferate and metastasize. Studies have shown that TME cells are not randomly distributed, but are more or less densely organized into different areas among epithelial cells, forming a complex background promoting tumor generation (4, 5). It was showed that TME cells dynamics in cancer seriously affect disease biology and may affect response to systemic therapy (5). In addition the interaction between TME and cancer cells could promote phenotypic heterogeneity, cell plasticity, and cancer cell stemness, improving tumor invasion and metastasis (6). Therefore, elucidating dynamic transcriptome changes in TME cells is important to identify mechanisms implicated in GC etiology.

While current transcriptome studies have identified TME variations in GC by bulk RNA-sequencing. For example, some literature showed the complex TME has severely weakened the efficacy of anti-tumor immunity (7–9). The infiltrating immunoinflammatory cells in the lamina propria of gastric mucosa displayed dynamic change during GC development (10). However, the principle of these analysis is based on the according to the assumption that every gene is equally expressed in every individual cell. Therefore, carrying out traditional RNA-sequencing is impossible to study the heterogeneity of TME cells at the subsets level.

Single-cell RNA transcriptome sequencing (scRNA-seq) is used to investigate cell heterogeneity and predicts and analyzes mutual cellular influences (11). The technique demonstrates good practicability for analyzing complicated cell environments and deciphering changes in cell conditions between multiple disease stages (12). Up to now, there has been a dearth of publication on this topic. Zhang et al. studied the characteristics of epithelial cells across different gastric diseases (NAG-CAG-IM-GC) (13). Sathe et al. compared GC with normal mucosa to identify cell reprogramming mechanisms in gastric TME (14). Wang et al. analyzed intratumoral heterogeneity of metastatic gastric adenocarcinoma (15). However, changes in TME cells during GC progression have not yet been elucidated, therefore, scRNA-seq could help determine the specific cellular and transcriptional features to distinguish the TME cells among the development of gastric diseases.

In this study, we generated a dynamic transcriptome map of several TME cell types during multi-stage disease comprising NAG-CAG-IM-GC processes using single-cell sequencing data. This map identified the multidimensional features of different TME cells during different disease states, including subclusters, marker genes, functional pathways, differentiation trajectories, activated transcription factors (TFs), immune checkpoints, and cell-cell communication patterns, etc. Our analyses revealed significantly increased heterogeneity of TME cells of macrophages, T, B, mast cell and fibroblast, endothelial, and pericyte cell during GC tumor formation, and has the potential to establish strategies for the early detection, diagnosis, and treatment of GC.

## Materials and Methods

### Data Acquisition

In total, 15 samples from 11 patients were analyzed in this study; three NAG, three CAG, six IM, and three GC samples (**Supplemental Table 1**). Data were downloaded from two sets of raw scRNA-seq data. Data, with the gene expression omnibus (GEO) accession number, GSE134520, comprising three NAG, three CAG, six IM and, one GC, were included. Another dataset with the database of Genotypes and Phenotypes (dbGaP) accession number, phs001818.v2 comprising two GC cases was included. These samples spanned the disease spectrum from gastritis to GC.

### Quality Control (QC) and scRNA-Seq Data Pre-Processing

The QC process was performed using Seurat (version 3.0.1) (16). A raw unique molecular identifier (UMI) count matrix was produced and converted into a Seurat object. The sequencing counts were negatively correlated with mitochondrial percentage levels and positively related to sequencing features (**Supplemental Figure 1A**). UMI counts from single cells whose UMI number was < 400, and the percentage of mitochondrial-derived UMI counts > 20 were deleted. To optimally eliminate potential doublets, single cells containing > 7000 genes were also filtered out. Then, using the “NormalizeData” function, single-cell gene expression data were normalized, and the normalization method was set to “LogNormalize”. Finally, we used the corrected expression matrix as an input for future studies.

### Dimensionality Reduction and Batch Effect Removal

We calculated the total number of UMI coding sequences per cell and genes in the samples (**Supplemental Figure 1B**). The results were initially summarized using principal component analysis (PCA). The top 20 principal components (PCs) and a resolution of 0.8 were selected by default using RunTSNE to reduce dimensionally. The “FindVariableFeatures” option in Seurat was used to calculate highly variable genes (HVG) (**Supplemental Figures 1C, D**). We applied the Harmony R package to eliminate the utility of batch among patients. The Adjusted Rand Index (ARI) was used to evaluate batch calibration based on the purity of cell types and the blend of batch (17). Low ARI scores indicated adequate mixing effects.

### Cell Type Recognition and t-Distributed Stochastic Neighbor Embedding (tSNE) Presentation

“FindAllMarkers” was used to identify differentially expressed genes (DEGs) in each cell type. We assigned cell types based on marker genes identified from previous studies (**Supplemental Table 2**). Single-cell clustering was visualized using tSNE analysis. The basic principle of this method was to re-calculate sample distances using the conditional probability of random neighbor fitting, which was based on Student t distributions in the high-dimensional space, so cells were in significantly separated clusters in the low-dimensional space.

### DEGs and Functional Enrichment Analysis

DEGs from cell clusters were identified using the “FindMarkers” function of Seurat. The following cutoff threshold values were used: adjusted p value (adj. *P* val) < 0.05 and logarithmic value (logFC) > 0.25. We used “FindMarkers” to evaluate DEGs from somatic cell clusters and loaded this information into clusterProfiler to perform gene ontology (GO) aggregation and GO enrichment analysis. The pathways of adj. *P* val was < 0.05 and was considered significantly enriched. The Retrieval of Interacting Genes database search tool for the retrieval of interacting genes (STRING; string-db.org) was used to assess interactive DEGs relationships, visualized using Cytoscape (18). Gene expression profiling interactive analysis (GEPIA; http://gepia. Cancer - pku. cn) is a web-based tool and provided customizable functions such as gene expression correlation analysis based on The Cancer Genome Atlas (TCGA) data (19).

Gene set variation analysis (GSVA, version 1.30.0) was performed using 50 hallmark gene-sets from the molecular signature database (20). To assess differential pathways between sub-cluster of cells, we calculated the activity scores using Limma package (version 3.38.3) (21). And then visualize T value data of the first 10 significantly different pathways (*P* < 0.05) using a heatmap containing the mean pathway scores of each cluster.

### Pseudotime Trajectory Analysis

Monocle 2 is a R package designed for single-cell trajectories (22) and was used to reveal changes in TME cells during multi-stage NAG-CAG-IM-GC processes. The following parameters were set: num_cells_expressed ≥ 10, mean expression ≥ 0.125, and q val < 0.01 (“differentialGeneTest” function). Trajectories were presented as tSNE plots, while dynamic expression heatmaps were built by the use of the “plot_pseudotime_heatmap” function.

### TF Analysis

Transcriptional activity among different cell clusters was assessed by SCENIC (version 1.1.0) (23) with the motif database of RcisTarget and GRNboost (corresponding to GENIE3 1.4.3, AUCell 1.4.1 and RcisTarget 1.2.1; with hg19:refseq-r80:10kb_up_and_down_tss.mc9nr). The area under the curve (AUC) of each module (calculated using SCENIC) was identified with the limma package. Regulons with an adj. *P* val <0.01 were considered for further investigation. Results were converted to binary data and visualized using the pheatmap function of R.

### Immune Checkpoint Analysis

We calculated the normalized expression levels of mean values of immune checkpoints of each cell cluster and normalized them into row Z scores to represent relative expression levels in different cell clusters.

### Cell-Cell Communication Analysis

CellChat is an open source R package (https://github.com/sqjin/CellChat) which infers, visualizes, and analyses intercellular communication data from scRNA-seq data (24). CellChatDataBase (http://www.cellchat.org/cellchatdb) contains 2,021 certified molecular structure interactions, including 60% of paracrine/autocrine data signal interactions, 21% of extracellular matrix-receptor interactions, and 19% of cell-cell contact interactions. To better predict and analyze intercellular communications, CellChat was used to identify differentially over-expressed ligands and receptors (L-Rs) for each cell cluster. Network visualization was performed in R.

### Immunofluorescence Staining

A total of 5 GC tissues and their corresponding adjacent IM and distal NAG tissue specimens were included, who were collected from the endoscopic submucosal dissection at Gastroenterology of China Medical University. The study was approved by the Ethics Committee of the First Affiliated Hospital of China Medical University. Formalin-fixed, paraffin-embedded Sections (4 mm) were deparaffinized in xylene and then hydrated in graded alcohol. EDTA (pH 8.0) was used for antigen retrieval in boiling water. The specimens were blocked by donkey serum (abs935, 1:20) for 30min. The following antibodies were used to detect specific fibroblast cell proteins: anti-RBP4 (abs136011, 1:100 dilution; Absin, China), anti-ABCA8 (NBP-91641,1:100 dilution; novus,US), anti-NPY (abs136011, 1:100 dilution; Absin, China), CST1 (abs136011, 1:100 dilution; Absin, China), and anti-ACTA2 (Kit-0006, MXB, China),was incubated for 1 hour at 37°C, rinsed in PBST, then detected by fluorescent secondary antibodies (Donkey anti-mouse IgG-AlexaFlour 594; 1:200; Absin, China) and Donkey anti-Rabbit IgG-AlexaFlour 488; 1:200; Absin, China) for 30 min at 37°C,rinsed in PBST, and finally stained with DAPI (abs42016321, 1:3000 dilution; Absin, China) for 10min and images captured on an Nikon ECLIPSE Ti2 inverted microscope.

## Results

### The Expression Profiling of TME Cells and Change Trends at Different Disease Stages

**Figure 1:**
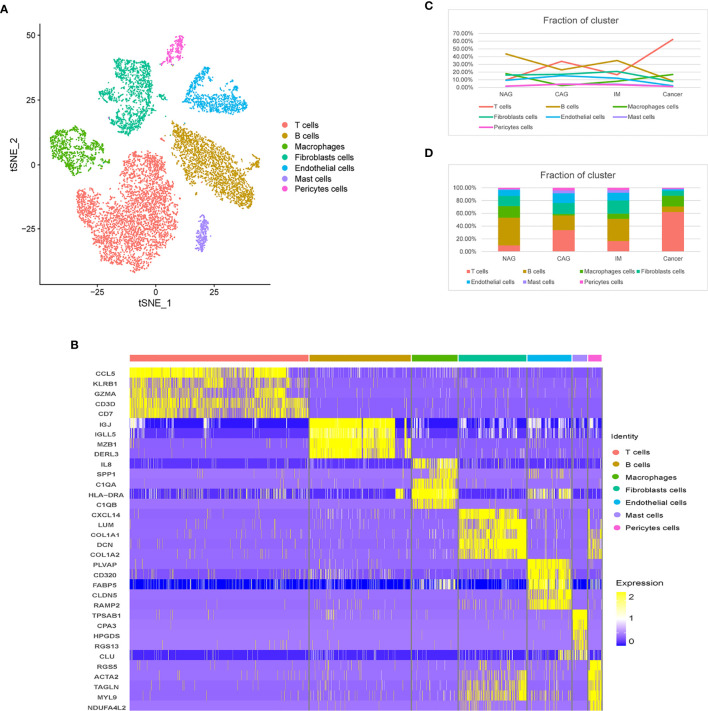
Distinct TME cell populations and expression signatures. **(A)** tSNE plot of TME cells based on their differential expression. **(B)** Heatmap plots showing the expression of the top five marker genes of seven major cell types identified in this profile. **(C)** Line chart showing the trend of the proportion of TME cells across the four pathological stages (NAG-CAG-IM-GC). **(D)** Stacked histogram showing TME cell composition across the four pathological stages.

To feature the single-cell general of gastric microenvironment, 66,063 single cells were obtained from NAG, CAG, IM, and GC stages. After QC, 45,336 cells remained. To identify distinct cell populations, we used the “method completed” option in Seurat to perform dimensionality reduction, eliminate batch effects, and develop an unsupervised module clustering (Method Details). As shown (**Supplemental Figure 1D**), when HVG = 3000 and ARI = 0.03740547, the batch effects among different samples were the lowest. Finally, we identified 24 main cell clusters along the GC cascade (**Supplemental Figures 1E, F**). Based on the expression of canonical markers, we excluded 13 epithelial cell clusters, 11 TME cell clusters were identified, including T cells (*CD2* and *CD3D*), B cells (*CD79A*), macrophages (*CSF1R* and CD68), mast cells (*TPSAB1*), fibroblasts (*DCN and PDPN)*, endothelial cells (ECs, marked as *VWF* and *ENG*), and pericytes (*RDGFRB* and *RGS5*) (**Figures 1A, B**). During progression along the NAG-CAG-IM-GC cascade, T cell proportions increased significantly, especially at the GC stage, and B cells and ECs decreased significantly. Macrophages, pericytes, mast cell, and fibroblasts exhibited slight fluctuations throughout the cascade (**Figures 1C, D**).

### The Dynamic Multidimensional Features of Macrophages in Different Disease States

**Figure 2:**
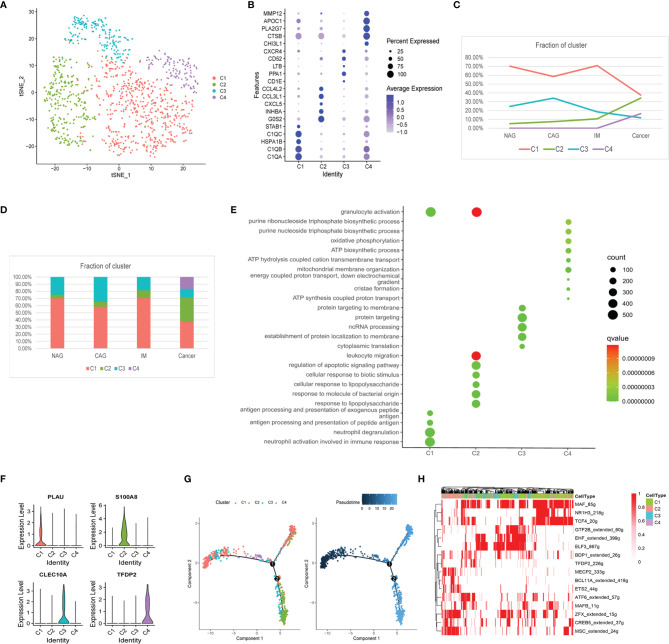
Changes in the composition of macrophages, gene expression, and functions at different stages. **(A)** tSNE plot of the four macrophage subclusters. **(B)** A bubble plot of the top five markers of each cell cluster; dot size represents abundance while the color represents expression level. **(C)** Line chart showing the trend of the proportion of the four clusters across the four stages. **(D)** Stacked histogram showing macrophage composition across the four stages. **(E)** Bubble plot showing the biological function of different cell clusters using GO; dot sizes represent abundance while the color represents q values. **(F)** Violin plots of marker genes in the four subclusters. **(G)** The differentiation trajectory of macrophages. Sections are color coded for pseudotime (right) and clusters (left). **(H)** AUC scores of TF expression regulation using SCENIC. Results converted to binary data were visualized as heatmap plots constructed using the pheatmap function of R.

We identified 1,162 macrophages in four subclusters (MacC1–MacC4) according to similar and differential gene expression (**Figure 2A**). From CAG to IM to GC, the proportion of MacC1 and MacC3 showed a downward trend, MacC2 was on the rise, whereas MacC4 was uniquely expressed at GC (**Figures 2C, D**). Marker genes and MacC1–C4 cluster functions were shown (**Figures 2B, E**). MacC1 comprised neutrophil activation and antigen presentation functions when compared with other cell clusters. MacC2 comprised granulocyte activation, leukocyte migration, and regulation of apoptotic signaling pathway functions. MacC3 cells were involved in protein localization at the membrane. MacC4 had a higher expression program for oxidative phosphorylation and ATP biosynthetic processes. We also identified new marker genes, *PLAU*, *S100A8*, *CLEC10A*, and *TFDP2* from MacC1 to MacC4, respectively (**Figure 2F**). Of these, *S100A8*, *CLEC10A*, and *TFDP2* exhibited significantly different expression profiles between cancer and precancerous stages (**Supplemental Figure 2A**). These results indicated that during NAG-CAG-IM-GC, macrophage clusters were involved in chemotaxis, antigen presentation, and apoptotic regulation. Similarly, macrophages were remodeled in the TME to participate in oxidative phosphorylation and ATP production to promote GC progression. This trajectory showed that MacC1 cells had the lowest pseudotime value (**Figure 2G**), of which MacC1 cells remained unchanged while some cells transformed to MacC2, processing through MacC3 to MacC4 cells. SCENIC analysis revealed that the TFs, MSC, MECP2, BCL11A, and ETS2 were up-regulated, whereas GTF2B, CREB5, MAF, NR1H3, and TCF4 were down-regulated during transformation (**Figure 2H**).

Furthermore, MacC2 cells showed elevated expression of chemokines (*CXCL5*, *CXCL2*, *CCL5*, *CCL3*, *CCL20*), interleukins (*IL8*, *IL6*, *IL1RN*, *IL1A*), and growth factors (*VEGFA*) when compared with other macrophage clusters, suggesting they interacted closely with other cells (**Supplemental Figure 2C**). Among them, for example, we found *CXCL5* expression gradually increased with GC development (**Supplemental Figure 2B**). Also, inhibitory checkpoint analysis showed that the MacC2 cluster exhibited higher expression of the genes, *ICOS*, *TNFRSF18*, *CD44*, *TNFSF14*, *TNFSF15*, and *CD48*, whereas MacC4 had high expression levels of *HAVCR2* (TIM3), *LAIR1*, *VSIR*, and *NRP1* when compared with other clusters (**Supplemental Figure 2D**). These data indicated that MacC2 and MacC4 macrophages had enhanced immunosuppressive properties.

Generally, macrophage phenotypes are labeled M1 or M2, with anti-cancer and cancer-promoting effects, respectively (17). We examined the expression of marker genes for M1 (*IL7R*, *IL2RA*, *BCL2A1*, *CXCL9*, and *CCL5*) and M2 (*CD163*, *CCL23*, *CCL13*, *CCL18*, and *MRC1*) across our macrophage clusters. However, M1/M2 gene expression could not distinguish between clusters (**Supplemental Figure 2E**), and these genes were co-expressed in the same cluster. These observations suggested macrophage gene expression heterogeneity during GC was irrelevant to M1/M2 classification.

### The Dynamic Multidimensional Features of T Cells at Different Stages

**Figure 3:**
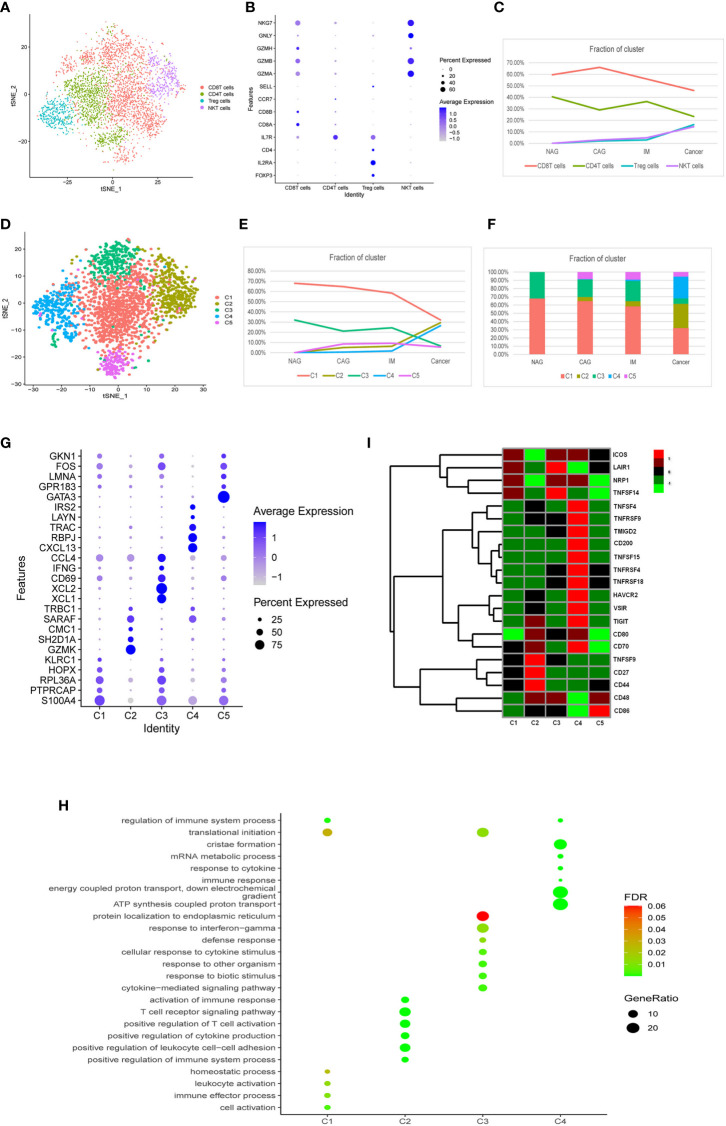
Characterization of multiple changes in T cell subtypes at different stages. **(A)** tSNE plots of 4,553 T cells, 2,410 CD8^+^T cells, 1,213 CD4^+^T cells, 478 Treg cells, and 452 NKT cells. Colors indicate cell type. **(B)** A bubble plot of markers of each cell type; dot sizes represent abundance while the color represents expression levels. **(C)** Line chart showing changing trend of the proportion of the four cell types across the four stages. **(D)** A tSNE plot of the five CD8^+^T cell subclusters. **(E)** Stacked histogram showing CD8^+^T composition across the four stages. **(F)** Line chart displaying changing trend of the proportion of the five clusters across the four stages. **(G)** Bubble plot of top five markers of CD8^+^ T cell clusters; dot sizes represent abundance while colors represent expression levels. **(H)** A bubble plot showing the biological functions of different cell clusters using GO. Dot sizes represent abundance while colors represent q values. **(I)** Heatmap of immune checkpoints altered during the differentiation process of CD8^+^T cells, which was clustered into five clusters. A row Z score was used to represent expression levels.

We identified four T cell clusters designated as CD8^+^T, CD4^+^T, Treg, and natural killer (NK) T cells according to known marker genes (**Figures 3A, B**). During the cascade from NAG to GC stages, CD8^+^T and CD4^+^T cells were gradually decreased and replaced by Treg and (NK)Tcells (**Figure 3C**). Approximately 1,213 CD4^+^T cells were represented by three subclasses (**Supplemental Figures 3A–C**). These classes did not significantly change during GC tumorigenesis (**Supplemental Figures 3D, E**), therefore, we conducted a subgroup analysis on CD8^+^T, Treg, and NKT cells.

As shown (**Figure 3D**), CD8^+^T (n = 2,410) cells were divided into five clusters (CD8^+^C1- CD8^+^C5). The proportion of CD8^+^C1 and CD8^+^C3 clusters gradually decreased, but CD8^+^C2 and CD8^+^C4 clusters gradually increased, especially from IM to GC where they displayed a sharp upward trend (**Figures 3E, F**). CD8^+^C5 did not change significantly between the different stages. Marker genes and functions of CD8^+^C1–CD8^+^C4 clusters are shown (**Figures 3G, H**). CD8^+^C1 displayed functions in immune effector processes, leukocyte activation, and translational initiation when compared with other clusters. CD8^+^C3 featured defense responses, responses to biotic stimulus, and cytokine-mediated signaling pathways. CD8^+^C2 exhibited functions toward the positive regulation of leukocyte cell-cell adhesion, T cell receptor signaling pathways, and immune responses. CD8^+^C4 exhibited substantially higher expression of the genes, *CXCL13, RBPJ, TRAC, LAYN*, and *IRS2* (**Figure 3I**), of which *CXCL13* and *LAYN* indicate T cell exhaustion (25). Notably, the inhibitory checkpoints genes, *TNFSF4, TNFRSF9, TMIGD2, CD200, TNFSF15, TNFRSF4, TNFRSF18, HAVCR2, VSIR, TIGIT*, and *CD70* were up-regulated in the CD8^+^C4 cluster (**Figure 3I**). These results indicated that between normal, precancerous, and cancer stages, a portion of CD8^+^T cells was exhausted and immunosuppressed, while another portion dominated the activation of immune responses to resist tumor cells.

**Figure 4:**
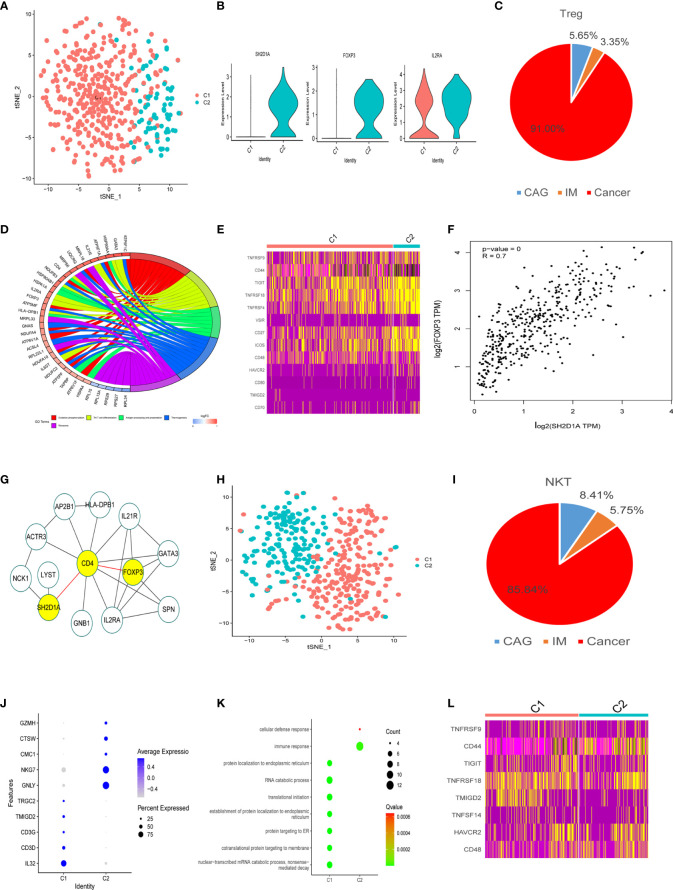
Treg and NKT cell clusters. **(A)** tSNE plot of two Treg cell subclusters. **(B)** Violin plots of marker genes for the C2 cluster. **(C)** A pie chart representing Treg cells from different pathological stages. **(D)** Circle diagram showing the functions of different cell clusters using GO, colors represent different functions, brighter colors indicate high expression. **(E)** Heatmap of immune checkpoints altered during the differentiation process of Treg cells. **(F)** GEPIA was used to assess the correlation of two genes based on the TCGA database. **(G)** STRING database was used to analyze protein interaction networks, while Cytoscape was used to visualize these networks. **(H)** tSNE plot of two NK cell subclusters. **(I)** Pie chart representing NK cells from different pathological stages. **(J)** Bubble plot of the top five markers in NK cell clusters; dot sizes represent abundance while colors represent expression levels. **(K)** Bubble plot showing the biological functions of different cell clusters using GO; dot sizes represent abundance while colors represent q values. **(L)** Heatmap of immune checkpoints altered during NK cell differentiation processes.

As shown (**Figure 4A**), 478 Treg cells were classified into two clusters (TreC1 and TreC2); TreC1 (FOXP3^-^IL2RA^+^) and TreC2 (FOXP3^+^IL2RA^+^) (**Figure 4B**), with most derived from the GC stage (**Figure 4C**). When compared with the TreC1 cluster, DEGs in the TreC2 cluster were enriched for oxidative phosphorylation and Th17 cell differentiation processes (**Figure 4D**). In addition, these cells expressed several immune checkpoints (*TIGIT, VSIR, HAVCR2, CD48, TMIGD2, CD80*, and *CD44*) and co-stimulatory molecules (*TNFRSF9, TNFRSF4, TNFRSF18, CD27, CD70*, and *ICOS*) (**Figure 4E**), suggesting important roles in carcinogenesis. Further analyses revealed that *SH2D1A* was a representative gene of the TreC2 cluster (**Figure 4B**). *FOXP3* and *SH2D1A* displayed a strong correlation (*P* < 0.001, R = 0.7, **Figure 4F**), however, no direct interactions were observed between them (**Figure 4G**).

We divided 452 NKT cells into two subtypes (C1 and C2), most of which came from tumor tissues (**Figures 4H, I**). Marker genes and their functions are shown (**Figures 4J, K**). The main function of C1 was related to protein localization to the endoplasmic reticulum, RNA catabolic processes, and translational initiation (**Figure 4K**) suggesting NKT cell activation. C2 was related to immune and cellular defense responses, indicating the potential for anti-tumor responses (**Figures 4K**). Some inhibitory molecules (*TIGIT*, *HAVCR2*, *TMIGD2*, *CD48*, *CD44*) and co-stimulatory molecules (*TNFRSF9*, *TNFRSF18*, *TNFSF14*) were also expressed in these clusters (**Figure 4L**).

### The Dynamic Multidimensional Features of B Cells at Different Stages

**Figure 5:**
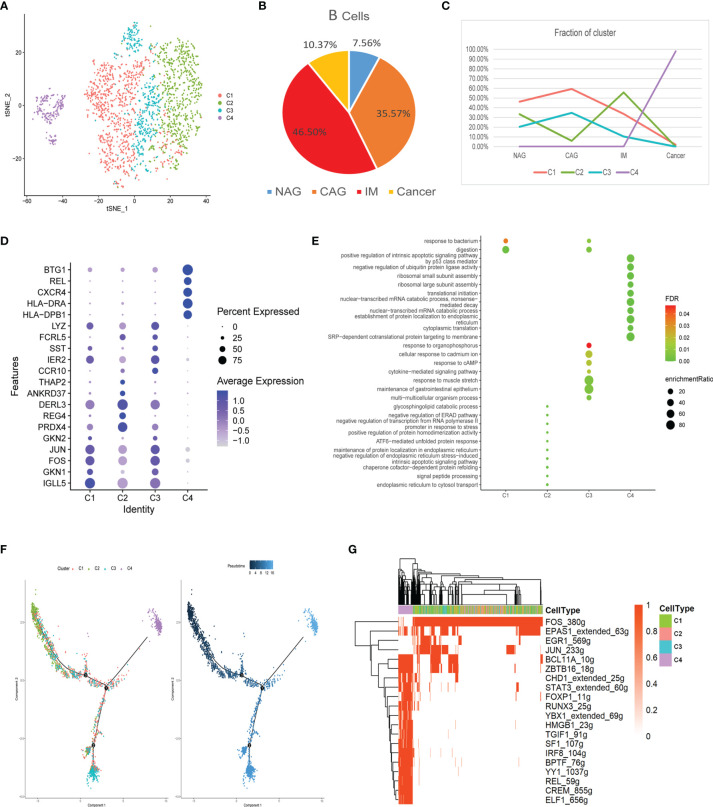
Characterization of multiple changes in B cell subtypes at different stages **(A)** tSNE plot of four B cell subclusters. **(B)** Pie chart representing B cells from different pathological stages. **(C)** Line chart displaying changing trend of the proportion of the four cell types across the four stages. **(D)** Bubble plot of the top five markers of each cell cluster; dot sizes represent abundance while colors represent expression levels. **(E)** Bubble plot showing the biological functions of different cell clusters using GO; dot sizes represent abundance while colors represent q values. **(F)** B cell differentiation trajectory in the four stages, with each color coded for pseudotime (right) and clusters (left). **(G)** AUC scores of transcription factor expression regulation using SCENIC. Results converted to binary data were visualized as heatmap plots constructed using the pheatmap function of R.

In total, 2,573 B cells were divided into four clusters (C1-C4), most of which were derived from the inflammation stage (**Figures 5A, B**). We showed that C1 and C3 clusters increased gradually in the CAG stage and then declined with disease progression (**Figure 5C**). The C2 cluster varied upwards and downwards. The C4 cluster only appeared in the GC stage. Marker genes and their functions are shown (**Figures 5D, E**). C1 was related to responses to bacteria and digestion functions. The C2 cluster comprised glycosphingolipid catabolic processes and processes involving the negative regulation of the endoplasmic reticulum-associated degradation (ERAD) pathway. C3 was related to cytokine-mediated signaling pathways and responses to cAMP and bacteria. The C4 cluster comprised functions related to the positive regulation of intrinsic apoptotic signaling pathways by p53 and the negative regulation of ubiquitin ligase activity. These results suggested that B cells actively participated in immune responses during inflammatory stages, but lost their functions at the cancer stage, with a tendency to induce apoptosis.

The trajectory showed that the C2 cell was the starting point that had the lowest pseudotime value. C3 and C4 appeared at each end of the differentiation trajectory (**Figure 5F**). SCENIC analysis showed that the activity of many key motifs, including those in STAT3, FOXP1, TGIF1, YY1, and REL was activated, whereas those in FOS, EPAS1, EGR1, and JUN were suppressed, which led to C2–C4 processes (**Figure 5G**).

### The Dynamic Multidimensional Features of Mast Cells at Different Stages

In total, 370 mast cells were divided into two clusters (C1 and C2). As shown (**Supplemental Figures 4A–C**), the MasC1 mast cell cluster was replaced by MasC2 in GC and was characterized by the high expression of *SLC18A2* and *HDC* genes.

### The Dynamic Multidimensional Features of Non-Immune Cells at Different Stages

Next, we assessed the transcriptome transition of non-immune cells in the TME, including fibroblast, endothelial, mast, and pericyte cells. As shown (**Supplemental Figures 4A–C**), the MasC1 mast cell cluster was replaced by MasC2 in GC and was characterized by the high expression of *SLC18A2* and *HDC* genes. As shown (**Supplemental Figures 4D–G**) the C1 cluster of pericyte cells had the highest composition ratio at every disease stage, therefore, we conducted a subgroup analysis of fibroblast and endothelial cells.

**Figure 6:**
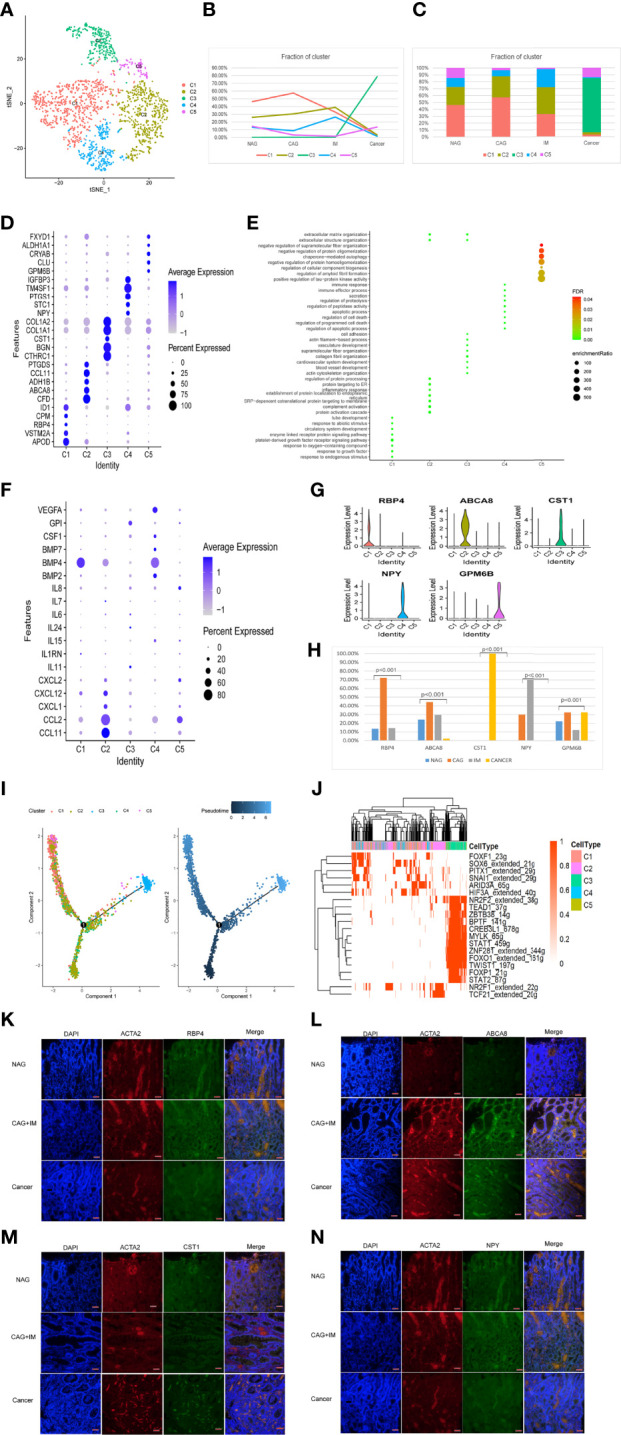
Identification of fibroblast clusters and expression features at different stages. **(A)** tSNE plot of five fibroblast cell subclusters. **(B)** Line chart displaying changing trend of the proportion of the five cell types across the four stages. **(C)** Stacked histogram showing fibroblast composition across the four stages. **(D)** Bubble plot of the top five markers of each cell cluster; dot sizes represent abundance while colors represent expression levels. **(E)** Bubble plot showing the biological functions of different cell clusters using GO; dot sizes represent abundance while colors represent q values. **(G)** Violin plots of marker genes in the five subclusters. **(F)** Bubble plot showing scale normalized expression of representative genes involved in chemokine, cytokine, and growth factor processes. **(H)** Histograms of scale normalized expression levels of three marker genes at each stage. **(I)** Fibroblast cell differentiation trajectory with each color coded for pseudotime (right) and clusters (left). **(J)** AUC scores of transcription factor expression regulation using SCENIC. Results converted to binary data were visualized as heatmap plots constructed using the pheatmap function of R. **(K)** Immunofluorescence staining of RBP4 (green) and ACTA2 (red) in different stages of stomach disease. Scale bars, 50 μm. **(L)** Immunofluorescence staining of ABCA8 (green) and ACTA2 (red) in different stages of stomach disease. Scale bars, 50 μm. **(M)** Immunofluorescence staining of CST1 (green) and ACTA2 (red) in different stages of stomach disease. Scale bars, 50 μm. **(N)** Immunofluorescence staining of NPY (green) and ACTA2 (red) in different stages of stomach disease. Scale bars, 50 μm.

In total, 1,730 fibroblasts cells were divided into five clusters (FibC1-C5) using different gene expression patterns (**Figure 6A**). FibC1 cluster cells dominated the CAG stage, whereas FibC2 and FibC4 clusters gradually increase towards the IM stage. The FibC3 cluster only appeared at the GC stage which may have been tumor-related, whereas FibC5 displayed a low composition ratio across all stages (**Figures 6B, C**). Marker genes and functions for all clusters are shown (**Figure 6D**). GO analyses indicated that responses to growth factors were highly enriched in FibC1 (**Figure 6E**), especially of BMP4 (**Figure 6F**). Inflammatory responses, complement activation, apoptosis, and proteolytic regulation processes were significantly higher in FibC2 and FibC4 (**Figure 6E**). Cardiovascular development, collagen fibril organization, and cell adhesion processes were highly enriched in FibC3 (**Figure 6E**). We also observed new marker genes (**Figure 6G**), of which *RBP4*, *ABCA8*, and *GPM6B* were primarily indented in the CAG stage; *CST1* mainly appeared at the GC stage and *NPY* dominated the IM stage (**Figure 6H**). To confirm the expression, immunofluorescence assays were performed as shown in **Figures 6K–N** that RBP4, S100A8, NPY, and CST1 were co-expressed with a conventional marker of fibroblasts (ACTA2) in different stages of stomach disease. RBP4, S100A8, NPY mostly appeared in CAG and IM stages, while CST1^+^ fibroblasts almost only appeared in the GC stage, and they were almost absent in NAG tissues.

Trajectory analysis showed that FibC2 cells had the lowest pseudotime value and that FibC3 appeared at the end of the differentiation trajectory, with the highest pseudotime value (**Figure 6I**). SCENIC analysis showed trajectory trend may be regulated by NR2F1, TCF21, FOXF1, and SOX6 expression decreased, whereas STAT1, STAT2, FOXP1, FOXO1, and NR2F2 were up-regulated (**Figure 6J**). The changes in TFs may be key to cell development mechanisms.

**Figure 7:**
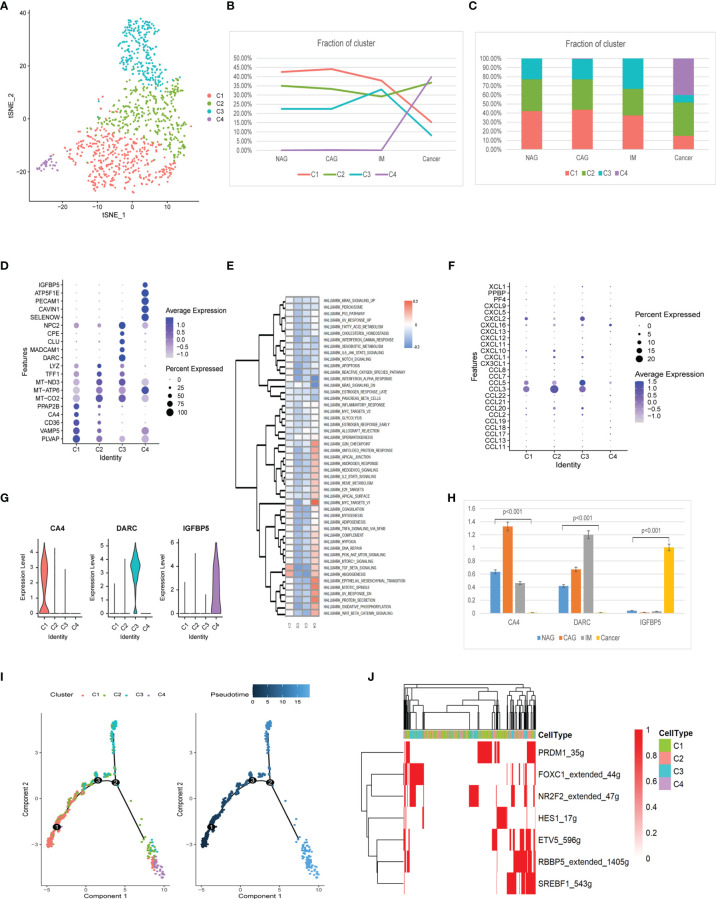
Identification of endothelial cell clusters and expression features at different stages. **(A)** tSNE plot of the four endothelial cell subclusters. **(B)** Line chart displaying changing trend of the proportion of the four cell types across the four stages. **(C)** Stacked histogram showing endothelium composition across the four stages. **(D)** Bubble plot of the top five markers of each cell cluster; dot sizes represent abundance while colors represent expression levels. **(E)** Heatmap showing activity differences in 50 hallmark pathways scored by GSVA. T values are calculated using a linear model. **(F)** Bubble plot showing scale normalized expression of representative genes involved in chemokine processes. **(G)** Violin plots of marker genes for the five subclusters. **(H)** Histograms of scale normalized expression levels of three marker genes at each stage. **(I)** Endothelial cell differentiation trajectory, with each color coded for pseudotime (right) and clusters (left). **(J)** AUC scores of TF expression regulation using SCENIC. Results converted to binary data were visualized as heatmap plots constructed using the pheatmap function of R.

Four endothelial cell clusters (EndC1-C4) were identified from 1,115 cells (**Figure 7A**). The EndC1 cluster gradually decreased during tumorigenesis. The EndC2 cluster was slightly increased in GC. EndC3 cluster cells were the majority cells at IM, whereas the EndC4 cluster only appeared at GC (**Figures 7B, C**). The EndC2 cluster had highly expressed mitochondrial genes and had no specific genes (**Figure 7D**). The EndC3 cluster exhibited negative correlations with TGF-β signaling, angiogenesis, and EMT (Epithelial-Mesenchymal Transition). The EndC4 cluster was mainly related to G2M checkpoint, MYC targets, and EMT processes (**Figure 7E**). Additionally, EndC4 displayed significantly increased chemokine expression (*CCL18, CCL3, CCL8, CXCL1, CXCL10, CXCL11, CXCL12, CXCL2, CXCL5, CXCL9*, and *PPBP*) (**Figure 7F**). The new marker gene, *CA4* primarily appeared at the CAG stage, whereas *DARC* figured in IM, and *IGFBP5* was mainly increased at the GC stage (**Figures 7G, H**).

Trajectory analysis showed that EndC1 cells had the lowest pseudotime value, whereas C3 and C4 clusters appeared at each end of the differentiation trajectory (**Figure 7I**). From EndC1–C3 processes, SCENIC analyses indicated the trajectory trend controlled by PRDM1, HES1 down-regulated expression, finally, FOXC1 and NR2F2 were up-regulated (**Figure 7J**). The C4 cluster did not activate the special motif modules.

### Constructing a TME-Epithelial Regulatory Network for IM/GC

**Figure 8:**
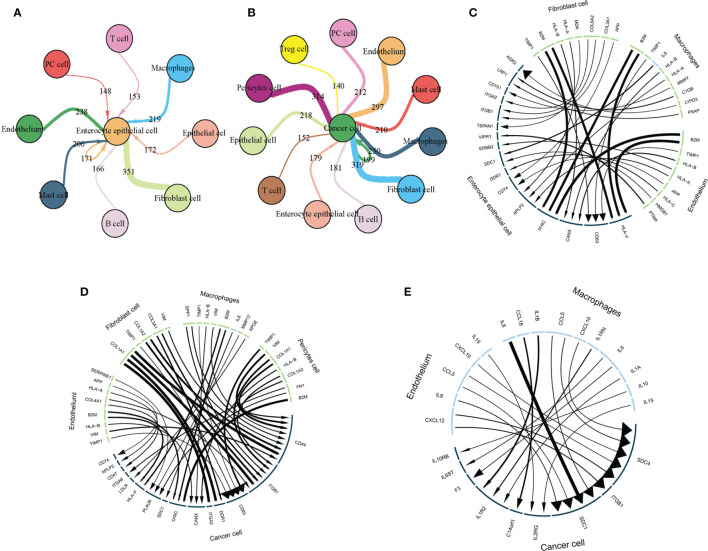
TME interacts with intestinal epithelial and cancer cells *via* L-R. **(A)** The TME interacts with intestinal epithelium cells *via* L-R interactions. Circle size represents cell counts, line and arrow size represents interaction counts; larger sizes reflect more counts and interactions with each other. **(B)** The TME interacts with cancer cells *via* L-Rs. Circle size represents cell counts and line size represents interaction counts. A larger size means more counts and interactions with each other. **(C)** The strongest 10 L-R pairs showing interactions between fibroblasts, macrophages, and endothelial cells with intestinal epithelial cells. Line and arrow size represents interaction counts. **(D)** The strongest 10 L-R pairs showing interactions between fibroblasts, macrophages, and endothelial cells with cancer cells. Line and arrow size represents interaction counts. **(E)** Macrophages and endothelial cells interact with cancer cells *via* cytokines. Line and arrow size represents interaction counts.

To further explore interactions between TME and epithelial cells, we used Cellchat to construct TME-epithelial networks at IM and GC stages. As shown (**Figure 8A**), macrophages, fibroblasts, and endothelial cells exerted the strongest effects on enterocyte epithelial cells at the IM stage. At GC, macrophages, fibroblasts, endothelial cells, and pericytes exerted the strongest effects (**Figure 8B**). In addition, we selected the 10 strongest L-R interactions in each cell cluster. At the IM stage (**Figure 8C**), the three TME cell groups all highly expressed *B2M*, which interacted with *TFRC* and *HLA-F* in enterocyte epithelial cells. However, at GC, the molecular interaction between TME and GC cells was altered, especially in fibroblasts. Highly-expressed *COL1A1*, *COL1A2*, and *COL3A1* in fibroblasts mainly interacted with *ITGA2*, *DDR1*, *ITGB1*, and *CD44* in GC cells (**Figure 8D**). Additionally, macrophages and endothelial cells secreted elevated cytokine levels at the GC stage, therefore, we analyzed interactions between these cytokines and GC cells. These data showed that cytokines primarily interacted with *SDC1*, *SDC4*, and *ITGB1* on the surface of GC cells (**Figure 8E**).

## Discussions

In this study, we reanalyzed the scRNA-seq data to describe the characteristics of TME cell clusters and showed the possible evolutionary trajectories of TME cells during the multistage process of GC development. And also, we investigated interactions between TME and epithelial cells. Overall, we depicted a dynamic transcriptome map of different TME cells during GC development.

Macrophages are a heterogeneous cell group with distinctive phenotypes and functions in complex microenvironments (26). Traditionally, these cells are divided into M1 (classically activated macrophage) and M2 types (alternatively activated macrophages) (27). However, the expression of M1/M2 genes did not distinguish clusters in our study, and these genes were co-expressed in the same cluster. This suggested that macrophage transcriptional heterogeneity was independent of the M1/M2 classification during GC. Our results also revealed four different macrophage types in TME, with each cluster showing dynamic changes at different stages. The MacC2 cluster showed an increasing trend towards GC progression and mainly participated in apoptotic signaling pathways. In addition, CXCL5 expression in MacC2 also specifically increased with disease development. Roca et al. observed that CXCL5 was transcriptionally unregulated in macrophages interacting with apoptotic cancer cells in contrast with noncancer cells during macrophage-driven efferocytosis, which accelerates inflammation and growth of prostate tumor metastases in bone (28). Some literature showed CXCL5-CXCR2-dominated cross-talk between cancer cells and macrophages could promote tumor metastases in gastric, hepatocellular, and prostate cancers (29, 30). This implied a critical role of macrophage-derived CXCL5 as a novel mechanism underlying tumor development and may be a viable target for cancer therapeutics. The MacC4 cluster only appeared at the GC stage and featured with the high expression of *CHI3L1* and *PLA2G7*. Chen et al. indicated that macrophage-secreted *CHI3L1* promoted GC metastasis *in vitro* and *in vivo* (28). In addition, Heng et al. showed that macrophage-derived *PLA2G7* was a novel tumor-promoting factor and was crucial in regulating tumor cell migration (31). The main function of the MacC4 cluster was related to oxidative phosphorylation and ATP biosynthetic processes. This character may fulfill the high energy and biosynthetic demands of tumor progression (32).

In addition, immune checkpoints like *CD48, CD44, TNFSF14*, and TNFRSF18 et al. were up-regulated in MacC2 clusters. Current studies have shown that *CD44, TNFRSF18*, and *TNFSF14*, which are highly expressed in macrophages, mainly promote the inflammatory response by activating the expression of the downstream pro-inflammatory cytokines (33–35). Meanwhile, *VSIR LAIR1* and *HAVCR2* were up-regulated in the MacC4 cluster. Some literature has indicated that the expression of the above immunosuppressive checkpoints in macrophages is mainly related to the induction of immune tolerance, promotion of macrophage polarization to furtherly improved tumor metastasis (36–38). Therefore, new therapies can be designed based on the above-mentioned immunosuppressive checkpoints targets to effectively prevent and inhibit tumor occurrence and metastasis.

S100A8 and TFDP2 were identified as new gene markers of macrophages and were significantly different between cancer and precancerous stages. Research has shown that S100A8 is produced by tumor-infiltrated inflammatory cells (39) that decorate the microenvironment and produce “pre-metastatic niches”, which benefit metastatic cell adhesion and growth (40). In addition, macrophages also secrete S100A8 to potentially promote tumor immune escape mechanisms (41). TFDP2 is a member of the dimerization partner (DP) family of TFs, which are primarily related to cell cycle regulation (42). Previous research showed that TFDP2 knock-down led to a significant reduction in the proliferation rate of erythropoiesis (43), but research into other cell processes is limited. Therefore, identifying specific macrophage subclusters using these marker genes may significantly benefit GC treatment strategies. However, the expression and functional roles of these genes in GC progression remain unknown and warrant further study. Trajectory analysis indicated that MacC2 cells had the highest pseudotime value with up-regulated TF of MECP2, BCL11A, and ETS2. Previous research showed that BCL11A regulates the development of lymphoid, erythroid, and dendritic cell lineages (44). ETS2 directly binds to regulatory sequences of *CCL3*, *CXCL5*, *CXCL10*, and immune cell recruitment mediators (45). How these TFs driving macrophage transformation and function require further study.

Four T cell subclusters were identified. CD8^+^C4 had a sharp upward trend from IM to GC. This cluster exhibited higher expression of *CXCL13* and *LAYN*, the marker of T cell exhaustion, which indicated a state of T cell dysfunction. Research has indicated T cell exhaustion is characterized by poor effector function, sustained expression of inhibitory receptors, and a transcriptional state distinct from that of functional effector or memory T cells (46), which may provide insights for immune-based cancer interventions (47).

Most Treg cells were expressed in tumor patients, including TreC1 (FOXP3^-^IL2RA^+^) and TreC2 (FOXP3^+^IL2RA^+^). Compared with the TreC1 cluster, TreC2 was primarily related to oxidative phosphorylation. Angelin et al. showed that Foxp3^+^Treg reprogramed T cell metabolism by inhibiting glycolysis and myc expression and enhancing oxidative phosphorylation. These adaptations permitted a metabolic advantage for Tregs in low glucose and lactic acid environments, thus they could resist the lactate-mediated inhibition of T cell function and proliferation (48). Also, this metabolic phenotype could explain how cancer cells evade immune destruction in the TME. Interestingly, we observed that SH2D1A was highly elevated in the TreC2. *SH2D1A* is one kind of X-linked lymphoproliferative (XLP) disease gene, whose loss-of-function mutations correlated with XLP. *SH2D1A* encodes the intracellular adaptor molecule SAP and interacts with signaling lymphocytic activation molecule (SLAM) family receptors by phosphorylating tyrosine residues (47). The *SH2D1A* gene mutation affects the production or expression of SAP, so that SAP cannot normally mediate the interaction between T/B cells. Thus, understanding the effects of Foxp3 and Sh2D1A may shed light on the metabolism and immunity regulation of Treg cell, which in turn have the potential to the translated into novel treatments for GC patients.

Additionally, we found most immune checkpoints are co-expressed in Treg and NKT cells, such as *TNFRSF9*, *TIGIT*, *TNFRSF18*, *TMIGD2*, *HAVCR2*, and *CD48*. The co-expression of these immune checkpoints may affect the occurrence and development of tumors. For example, Fourcade et al. reported that TIGIT^+^ Tregs were highly suppressive, stable, and enriched in tumors, whereas blockaded TIGIT counteracted Treg suppression in patients with melanoma (49). In addition, blockaded TIGIT prevented NKT cell exhaustion, and promoted NKT cell-dependent tumor immunity in several tumors (50, 51). However, the co-expression of other immune checkpoints has not been reported. We proposed select immune checkpoint inhibitors with dual expression of Treg and NKT cells may provide a new strategy for cancer therapy.

Four B cell subclusters were identified in this study. The C2 cluster mainly appeared at the IM stage and was related to glycosphingolipid catabolic processes and negative regulation of the ERAD pathway. But the relationship between this function of B cells and IM has not yet been reported. The C4 cluster only appeared at the GC stage and tended to positively regulate the intrinsic apoptotic signaling pathway *via* p53 and the negative regulation of ubiquitin ligase activity. Therefore, this cluster of B cell may exert anti-cancer effects by apoptosis in cancer cells. Trajectory data showed that C2 cells were starting points as they had the lowest pseudotime value, whereas C3 and C4 appeared at each end of the differentiation trajectory. SCENIC analyses showed that up-regulated STAT3, FOXP1, RUNX3, and REL may be responsible for functional changes and the terminal differentiation of B clusters. A previous study showed that STAT3 expression promoted B cell proliferation and differentiation (52). FOXP1 and RUNX3 also control mature B cell survival and maturation (53, 54) and *Rel* is expressed in B cells and reticulocytes and is essential for proliferation, survival, and antibody production (55). Activation of these TFs suggested B cell differentiation and maturation, however, further exploration of these TF regulatory roles will help clarify C4 cluster mechanisms in GC.

Mast cells were divided into two clusters. During dynamic disease processes, MasC2 cells gradually increased in the function of production and release of histamine and other transmitters. The effect of histamine released by mast cells in promoting the conversion of malignant tumor capillaries was comprehensively analyzed (56). Therefore, this cluster of mast cells may be crucial for GC progression.

Similar to immune cells, non-immune-related TME cells also promoted GC progression. We examined transcriptomic alterations of fibroblast, endothelial, and pericyte cells. Fibroblasts were divided into five clusters. FibC4 mainly appeared at the IM stage, and its main functions were related to secretion, the regulation of apoptotic processes, and peptidase activity. FibC4 was characterized by the high expression of *IGFBP3* which promoted both fibroblasts into myofibroblasts, and also matrix remodeling (57). Xu et al. showed that myofibroblasts metabolized proteoglycans containing laminin and induced hepatocyte-to-ductal metaplasia based on αvβ6 integrin-induced (58). Thus, we propose that inhibiting *IGFBP3* expression and reducing myofibroblast transformation may be key to preventing IM. As tumor-related fibroblasts, FibC3 cells exhibited cell adhesion, vasculature development, and actin cytoskeleton organization functions, indicating a close relationship with tumor metastasis. These cells also displayed high *CTHRC1* levels, which were shown to promote tumorigenesis, proliferation, invasion, and metastasis in several malignant tumors *via* different signaling pathways (59). We also found several new fibroblast marker genes and verify their expression in fibroblasts at different stages of gastric disease. *CST1*, as a cystatin superfamily that encompasses proteins that contain multiple cystatin-like sequences, may have correlations with fibroblasts cell activation (60). *NPY* encodes a neuropeptide, inhibiting its expression leads to increased cancer cell apoptosis, decreased exercise capacity, and changes in energy metabolism pathways (61). In addition, *ABCA8* and *RBP4* are mainly related to lipid metabolism and transportation (62, 63). However, the expression and function of these mark gene in fibroblasts during the multi-step development of GC has not been comprehensively reported. The dynamic changes in fibroblast clusters observed here suggested that at IM and GC stages, fibroblasts reshaped the immune microenvironment and promoted GC metaplasia and development.

Endothelial cells were divided into four clusters. The EndC3 cluster was the major component of IM and exhibited mainly negative correlations with TGF-β signaling, angiogenesis, and EMT. Previous studies suggested that decrease TGF-β signaling was related to atrophic gastritis (64, 65). In addition, EndC3 was characterized by the specific expression of *DARC*. *DARC* is a seven-transmembrane G protein-coupled reactive protein kinase found in blood cells and the surface layer of endothelial cells lining venules behind capillaries (66). It was recently confirmed as being expressed on lymph node fibroblasts (66). *DARC* functions as a decoy receptor for a variety of CXC and CC chemokines, including those with pro-malignant and pro-inflammatory roles (67). Therefore, EndC3 appears under inflammatory stimulation conditions, its main role may be to physically remove inflammatory factors in the extracellular environment to reduce the inflammatory response. However, its mechanistic role in IM requires further investigation. EndC4 is mainly expressed in the tumors stage the main function of MYC targeting and EMT protein secretion signatures, suggesting this cluster exhibited a high proliferation phenotype and was crucial to GC genesis and progression. *IGFBP5* is a new marker gene identified in this cluster; it is a secreted protein related to cell adhesion, proliferation, migration, inflammatory mediators, and fibrosis (68). A recent study reported that *IGFBP5* was highly expressed in a variety of cancers, promoted cancer occurrence and development, and was implicated in radiotherapy and chemotherapy resistance (69), but its significance in endothelial cells warrants further research. These data indicated that functional changes in endothelial cells were strongly correlated with GC progression.

Pericytes are parietal cells in blood capillaries and were recently identified as regulating the production and function of capillary shape during development (70). When compared with other TME cells, little is known about pericyte identification, recruitment, and interactions with tumor or other stromal cells. Thus, they may have the potential to be underlying stromal targets for cancer treatment. While we classified pericytes and analyzed their functions, their precise role in GC genesis and progression requires further investigation.

The CellChat analysis indicated that macrophages, fibroblasts, and endothelial cells were crucial for the genesis and progression of the IM and GC stages. At the IM stage, high *B2M* expression levels in TME cells interacted with intestinal epithelial cells *via TFRC* and *HLA-F.* Wang et al. reported that *TFRC* is a major inducer of ferroptosis, the expression of which indicates iron uptake and storage dysfunction (70). The ferroptosis process is iron-dependent and is characterized by increased lipid active oxygen (71). A recent study showed that a high-lard diet induced IM (72). Therefore, the accumulation of lipid active oxygen induced by ferroptosis may be an important IM mechanism at the gastric epithelium, hence, preventing ferroptosis in epithelial cells may be effective in preventing gastric epithelial IM. At the GC stage, interactions between the TME and epithelial cells changed significantly, especially for fibrocytes. *COL1A1, COL1A2*, and *COL3A1* expressed by fibroblasts interacted with cancer cells by *ITGA2, DDR1*, and *ITGB1*. Studies have shown that *ITGA2*, *DDR1*, and *ITGB1* were strongly correlated with GC genesis, development, and metastasis (73–76). Therefore, fibroblasts may be crucial for these processes. However, the molecular mechanisms underpinning their interactions require greater elucidation. In addition, macrophages and endothelial cells secreted large numbers of cytokines which interacted with *SDC1, SDC4*, and *ITGB1* in cancer cells. Studies have shown that *SDC1* and *SDC4* led to EMT activation and further promoted GC metastasis (77, 78), therefore, preventing these interactions could inhibit GC metastasis.

Although several important findings were generated in this study, we acknowledge some limitations. Firstly, while we attempted to identify most TME cells, some cell types were not found, therefore, we must increase patient sample numbers to solve this issue. Secondly, although we identified the transformation of the TME as the key element in the development of gastric diseases, the underlying molecular mechanisms require further investigation.

In conclusion, our comprehensive characterization of the TME at different cellular stages revealed dynamic changes in TME cells, from inflammation to cancer. We observed a set of key transition markers which were related to the carcinogenic evolution of TME cells, and we delineated landmark dynamic carcinogenic trajectories of TME cells. We identified three TME cell groups which were strongly correlated with IM and GC occurrence *via* cell-cell interactions. Our results also indicated the phenotypic convergence of various TME cell types in whole tumor formation processes during GC. Equally, we have invaluable molecular evidence for GC early detection, diagnosis, and treatment strategies.

## Data Availability Statement

Publicly available datasets were analyzed in this study. This data can be found here: GEO: GSE134520 and stanford medicine (https://dna-discovery.stanford.edu/research/datasets/).

## Ethics Statement

The studies involving human participants were reviewed and approved by the Ethics Committee of the First Affiliated Hospital of China Medical University. The patients/participants provided their written informed consent to participate in this study. Written informed consent was obtained from the individual(s) for the publication of any potentially identifiable images or data included in this article.

## Author Contributions

YY and YG conceived the study. HY, YG, and YY drafted the manuscript and performed the analysis. HY, HZ, and SL performed the literature search and collected the data. HY and RG contributed to drafting the manuscript and interpreting data. All authors contributed to the article and approved the submitted version.

## Funding

This work was supported by the National Natural Science Foundation of China (Award No. 81970501) and the National Key R&D Program of China (Grant 2018YFC1311600).

## Conflict of Interest

The authors declare that the research was conducted in the absence of any commercial or financial relationships that could be construed as a potential conflict of interest.

## Publisher’s Note

All claims expressed in this article are solely those of the authors and do not necessarily represent those of their affiliated organizations, or those of the publisher, the editors and the reviewers. Any product that may be evaluated in this article, or claim that may be made by its manufacturer, is not guaranteed or endorsed by the publisher.

## Supplementary Material

Supplementary Figure 1 Distribution of gene numbers and gene expression profiles of all single-cell sequencing data, dimensionality reduction, and elimination of batch effects. **(A)** The relationship between the percentage of mitochondrial genes and mRNA reads, and the relationship between mRNA levels. **(B)** Scatterplot illustrating UMI, number of genes, and the percentage of mitochondrial genes in each cell at the four stages. **(C)** Before removing batch effect between batches; 2000 variable features (left), 3000 variable features (middle), and 4000 variable features (right) were used, respectively. Batch effects were evaluated by calculating ARI. **(D)** After removing batch effects between batches; 2000 variable features (left), 3000 variable features (middle), and 4000 variable features (right) were used, respectively. Batch effects were evaluated by calculating ARI. **(E)** The t-SNE plot of 45,336 high-quality cells to 24 clusters. **(F)** tSNE plot of all the single cells, with each color coded for 15 major cell types. **(G)** tSNE plot of the source of the 24 subclusters of cells.

Supplementary Figure 2 Macrophage changes in gene expression in different gastric diseases. **(A)** Histograms of scale normalized expression levels of four marker genes at each stage. **(B)** Differentiation trajectory analysis of CXCL5 expression. The abscissa represents pseudotime, the ordinate represents gene expression, and dots with different colors represent different macrophage clusters. **(C)** Bubble plot showing scale normalized expression of representative genes involved in chemokine, interleukin, and growth factor processes. **(D)** Heatmap of immune checkpoints altered in the differentiation process of macrophages, which was clustered into four clusters. A row Z score was used to represent expression levels. **(E)** Heatmap showing expression of M1/M2 genes from each macrophage cluster.

Supplementary Figure 3 Clusters of CD4^+^T cells in different gastric diseases. **(A)** tSNE plot of three CD4^+^T cell subclusters. **(B)** Bubble plot of the top five markers of each cell cluster; dot sizes represent abundance while colors represent expression levels. **(C)** Bubble plot showing scale normalized expression of representative genes involved in cytokine and growth factor processes. Dot sizes represent a percentage of cells expressing corresponding genes, while colors represent gene expression levels in clusters. **(D)** Stacked histogram showing CD4^+^T composition across the four stages. **(E)** Line chart displaying changing trend of the proportion of the three cell clusters across the four stages.

Supplementary Figure 4 Identification of mast and pericyte cell clusters and expression features. **(A)** tSNE plot of the two mast cell subclusters. **(B)** Line chart displaying changing trend of the proportion of the two cell clusters across the four stages. **(C)** Violin plots of genes featured in the C2 cluster. **(D)** tSNE plot of the two pericyte subclusters. **(E)** Line chart displaying changing trend of the proportion of the two cell clusters across the four stages. **(F)** Bubble plot of the top five markers of each cell cluster; dot sizes represent abundance while colors represent expression levels. **(G)** Bubble plot showing the biological functions of different cell clusters using GO, dot sizes represent abundance while colors represent q values.

Supplementary Table 1 The clinical features of patient cohort used in the study.
