# Supplementary figures and images for: A Dynamic Transcriptome Map of Different Tissue Microenvironment Cells Identified During Gastric Cancer Development Using Single-Cell RNA Sequencing
Source: Front Immunol. 2021 Oct 21;12:728169. doi: 10.3389/fimmu.2021.728169 (PMC8566821; doi:10.3389/fimmu.2021.728169)

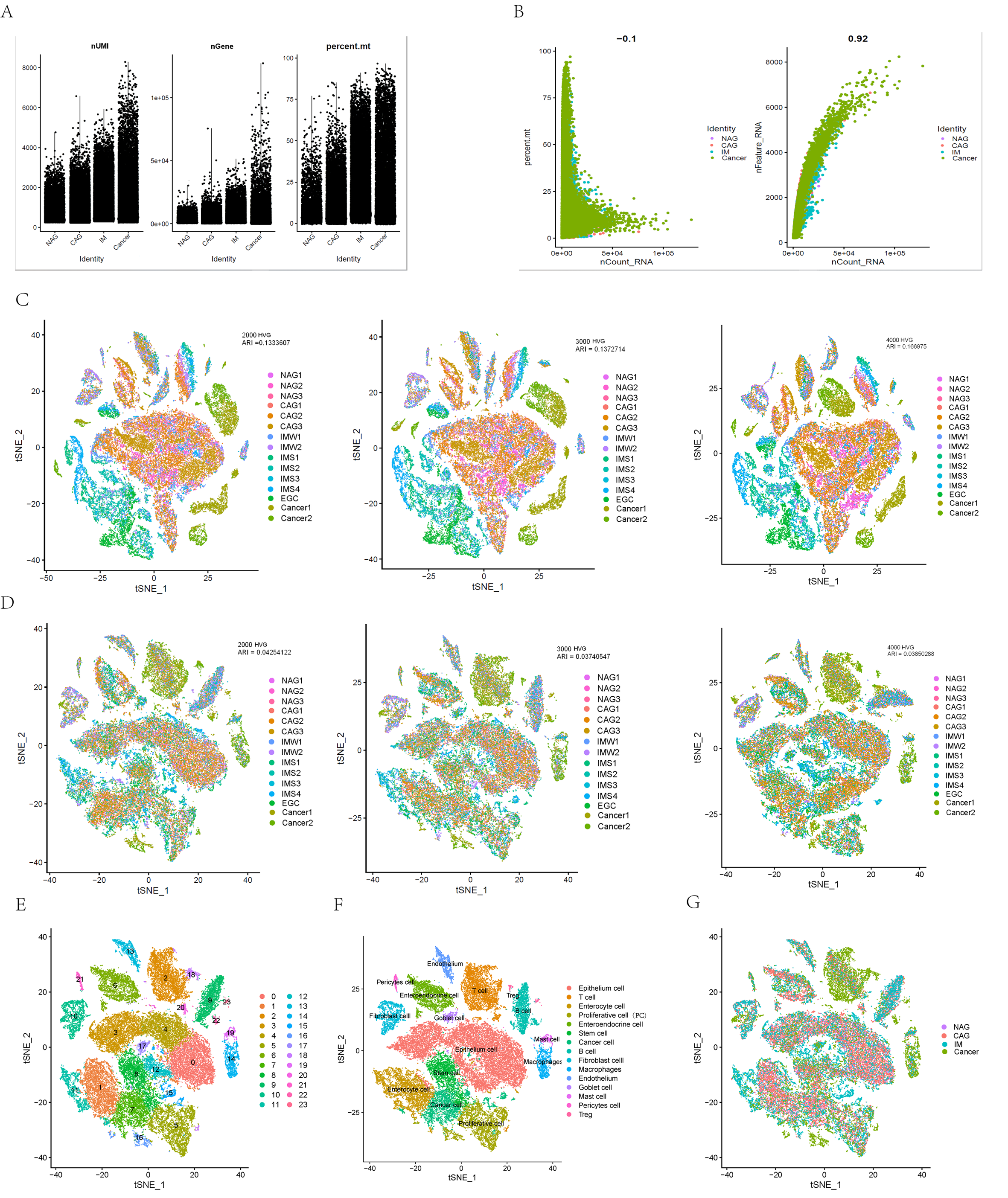

Supplement: Supplementary Figure 1 — Distribution of gene numbers and gene expression profiles of all single-cell sequencing data, dimensionality reduction, and elimination of batch effects. (A) The relationship between the percentage of mitochondrial genes and mRNA reads, and the relationship between mRNA levels. (B) Scatterplot illustrating UMI, number of genes, and the percentage of mitochondrial genes in each cell at the four stages. (C) Before removing batch effect between batches; 2000 variable features (left), 3000 variable features (middle), and 4000 variable features (right) were used, respectively. Batch effects were evaluated by calculating ARI. (D) After removing batch effects between batches; 2000 variable features (left), 3000 variable features (middle), and 4000 variable features (right) were used, respectively. Batch effects were evaluated by calculating ARI. (E) The t-SNE plot of 45,336 high-quality cells to 24 clusters. (F) tSNE plot of all the single cells, with each color coded for 15 major cell types. (G) tSNE plot of the source of the 24 subclusters of cells. [file Image_1.tif]

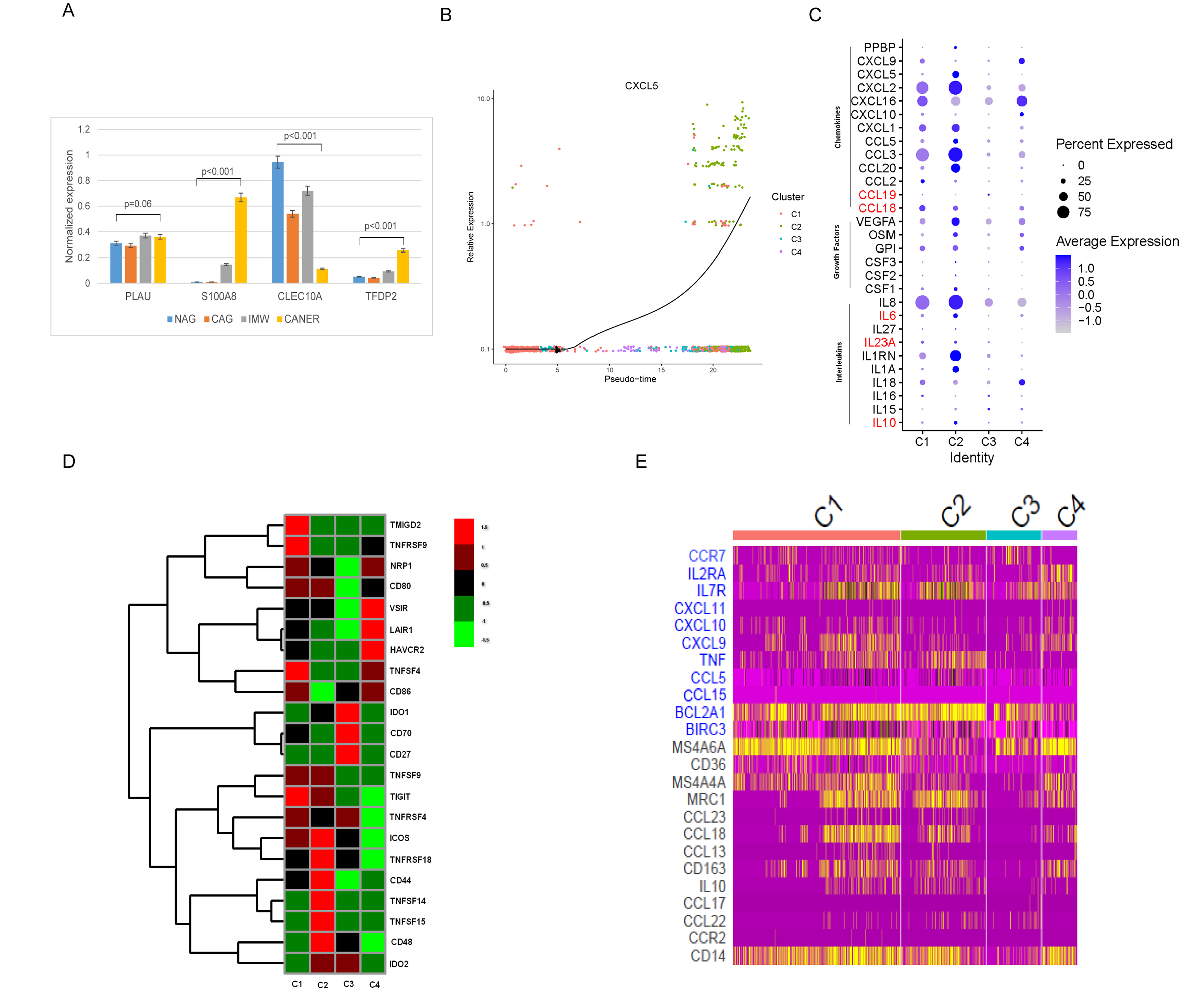

Supplement: Supplementary Figure 2 — Macrophage changes in gene expression in different gastric diseases. (A) Histograms of scale normalized expression levels of four marker genes at each stage. (B) Differentiation trajectory analysis of CXCL5 expression. The abscissa represents pseudotime, the ordinate represents gene expression, and dots with different colors represent different macrophage clusters. (C) Bubble plot showing scale normalized expression of representative genes involved in chemokine, interleukin, and growth factor processes. (D) Heatmap of immune checkpoints altered in the differentiation process of macrophages, which was clustered into four clusters. A row Z score was used to represent expression levels. (E) Heatmap showing expression of M1/M2 genes from each macrophage cluster. [file Image_2.tif]

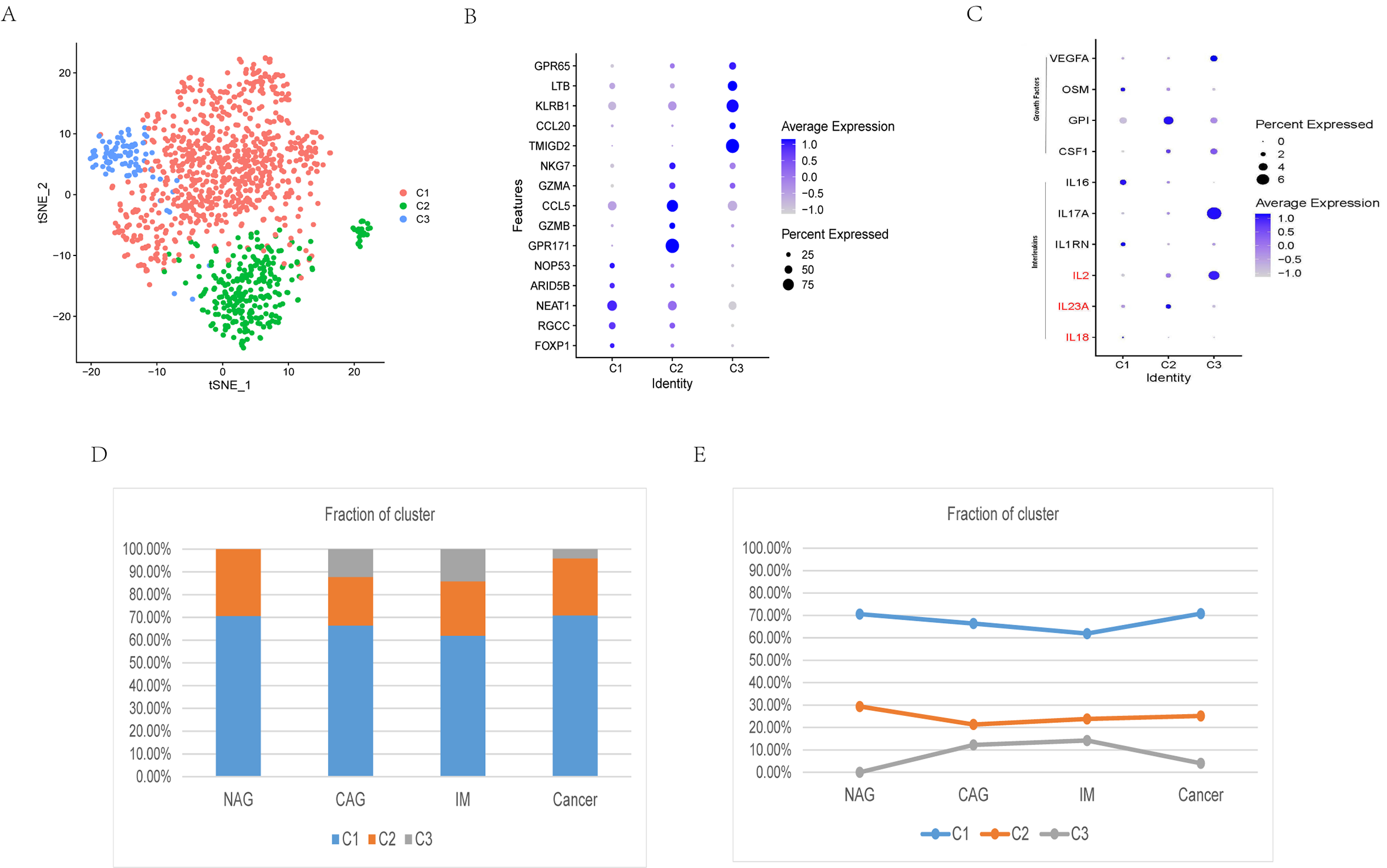

Supplement: Supplementary Figure 3 — Clusters of CD4+T cells in different gastric diseases. (A) tSNE plot of three CD4+T cell subclusters. (B) Bubble plot of the top five markers of each cell cluster; dot sizes represent abundance while colors represent expression levels. (C) Bubble plot showing scale normalized expression of representative genes involved in cytokine and growth factor processes. Dot sizes represent a percentage of cells expressing corresponding genes, while colors represent gene expression levels in clusters. (D) Stacked histogram showing CD4+T composition across the four stages. (E) Line chart displaying changing trend of the proportion of the three cell clusters across the four stages. [file Image_3.tif]

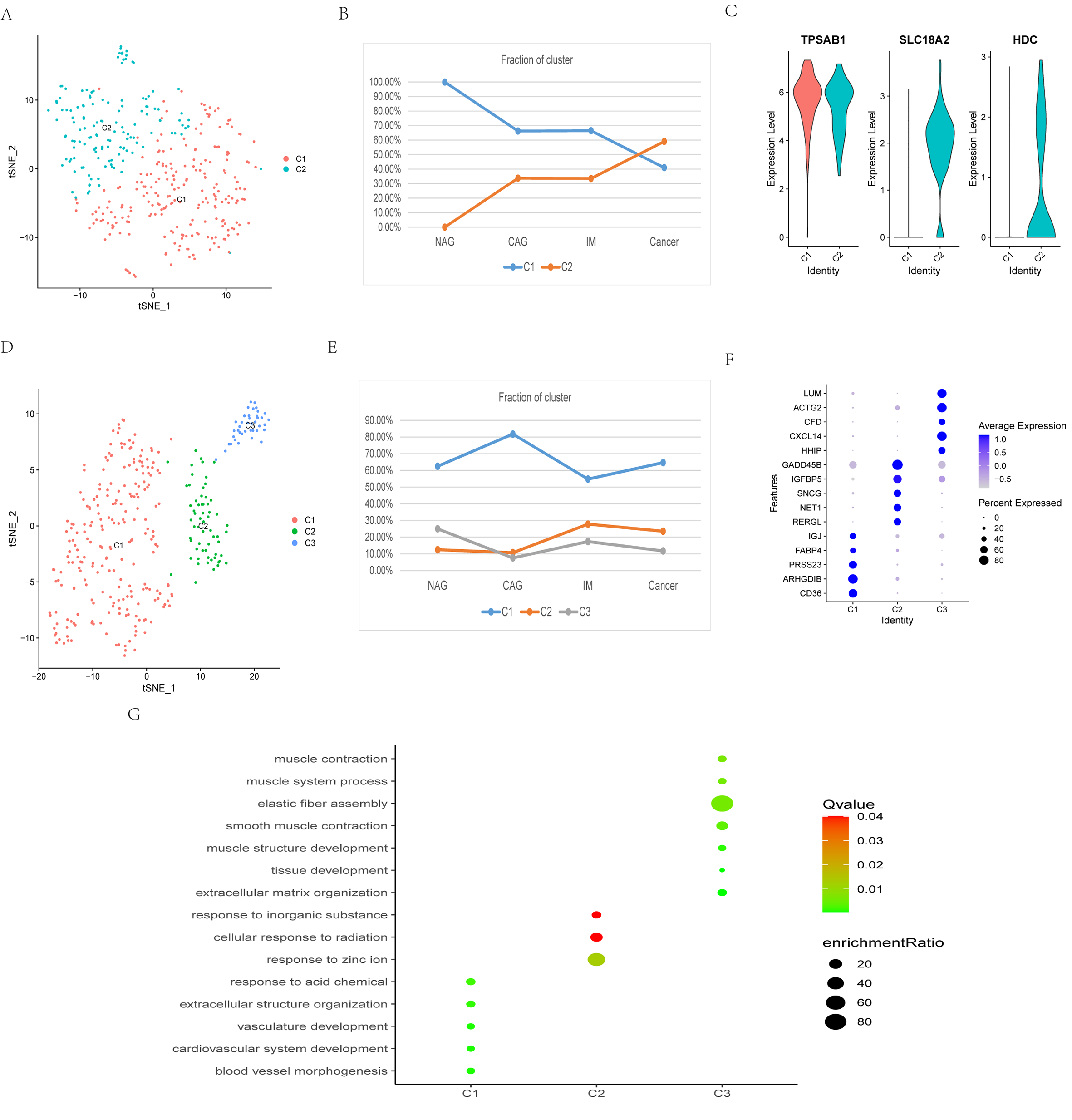

Supplement: Supplementary Figure 4 — Identification of mast and pericyte cell clusters and expression features. (A) tSNE plot of the two mast cell subclusters. (B) Line chart displaying changing trend of the proportion of the two cell clusters across the four stages. (C) Violin plots of genes featured in the C2 cluster. (D) tSNE plot of the two pericyte subclusters. (E) Line chart displaying changing trend of the proportion of the two cell clusters across the four stages. (F) Bubble plot of the top five markers of each cell cluster; dot sizes represent abundance while colors represent expression levels. (G) Bubble plot showing the biological functions of different cell clusters using GO, dot sizes represent abundance while colors represent q values. [file Image_4.tif]
