# Supplementary material for: A Dynamic Transcriptome Map of Different Tissue Microenvironment Cells Identified During Gastric Cancer Development Using Single-Cell RNA Sequencing
Source: Front Immunol. 2021 Oct 21;12:728169. doi: 10.3389/fimmu.2021.728169 (PMC8566821; doi:10.3389/fimmu.2021.728169)
Supplement: Supplementary Table 1 — The clinical features of patient cohort used in the study. [file Table_1.docx]

Supplemental Table 1: The clinical features of patient cohort used in the study

| **Patient ID** | **Age** | **Sex** | **Diagnosis** | **Histological type** |
| --- | --- | --- | --- | --- |
| NAG1 | 58 | M | NAG | NAG |
| NAG2 | 56 | F | NAG | NAG |
| NAG3 | 62 | M | NAG | NAG |
| CAG1 | 51 | M | CAG | CAG |
| CAG2 | 62 | F | CAG | CAG |
| CAG3 | 62 | F | CAG | CAG |
| IMW1 | 63 | M | IM | IM-W (incomplete IM) |
| IMW2 | 48 | F | IM | IM-W (incomplete IM) |
| IMS1 | 68 | M | IM | IM-S (incomplete IM) |
| IMS2 | 68 | M | IM | IM-S (incomplete IM) |
| IMS3 | 67 | M | IM | IM-S (incomplete IM) |
| IMS4 | 67 | M | IM | IM-S (incomplete IM) |
| EGC | 67 | M | GC | Early intestinal adenocarcinoma |
| 5931 | 75 | M | GC | Intestinal adenocarcinoma |
| 6207 | 70 | F | GC | Intestinal adenocarcinoma |

* NAG, No Atrophic Gastritis, CAG, Chronic Atrophic Gastritis, IM-W, Intestinal metaplasia with

wild level; IM-S, Intestinal metaplasia with severe level; EGC, Early Gastric Cancer
