# Supplementary material for: A Dynamic Transcriptome Map of Different Tissue Microenvironment Cells Identified During Gastric Cancer Development Using Single-Cell RNA Sequencing
Source: Front Immunol. 2021 Oct 21;12:728169. doi: 10.3389/fimmu.2021.728169 (PMC8566821; doi:10.3389/fimmu.2021.728169)
Supplement: Supplementary file 6 [file Table_2.docx]

**Supplemental Table 2: Marker genes in stomach used to define the cell lineage with literature references**

| **Gene** | **Marker_of** | **Reference (DOI/PMID)** |
| --- | --- | --- |
| MUC5AC | Gastric epithelium | 10.1053/j.gastro.2014.09.042 |
| PGC | Gastric epithelium | 10.1053/j.gastro.2014.09.043 |
| MUC6 | Gastric epithelium | 10.1053/j.gastro.2014.09.045 |
| TFF1 | Gastric epithelium | 10.1053/j.gastro.2014.09.046 |
| TFF2 | Gastric epithelium | 10.1053/j.gastro.2014.09.047 |
| MUC2 | Intestinal epithelium | 10.1053/j.gastro.2014.09.050 |
| FABP1 | Intestinal epithelium | 10.1002/pro.3875 |
| CHGA | enteroendocrine cell（Intestinal cell) | PMID: 10461347 |
| CHGB | enteroendocrine cell（Intestinal cell) | PMID: 10461347 |
| GAST | Gastric neuroendocrine cells | PMID: 10461347 |
| SST | Gastric neuroendocrine cells | PMID: 10461347 |
| TFF3 | Gastric adenocarcinoma cells | 10.7150/ijms.5500 |
| EPCAM | Gastric adenocarcinoma cells | 10.1007/s00432-009-0569-5 |
| LGR5 | Gastric stem cells | 10.1053/j.gastro.2014.09.053 |
| OLFM4 | Gastric stem cells | 10.1053/j.gastro.2014.09.053 |
| MKI67 | PC (proliferative) | 10.1007/s00412-018-0659-8 |
| BIRC5 | PC (proliferative) | 10.1242/jcs.223826 |
| CD163 | Macrophages | 10.1186/s40425-017-0215-8 |
| CD68 | Macrophages | 10.1186/s40425-017-0215-9 |
| FCGR3A | Macrophages | 10.1038/ncomms14049 |
| CD3D | T-cells | 10.1186/s40425-017-0215-14 |
| CD3E | T-cells | 10.1186/s40425-017-0215-15 |
| CD3G | T-cells | 10.1186/s40425-017-0215-16 |
| CD8A | CD8 T cells | 10.1186/s40425-017-0215-8 |
| CD8B | CD8 T cells | 10.1186/s40425-017-0215-8 |
| CD4 | CD4 T cells | 10.1016/bs.ircmb.2018.05.007 |
| IL7R | CD4 T cells | 10.1038/s41590-019-0479-x |
| FOXP3 | Treg | 10.1186/s40425-017-0215-17 |
| IL2RA | Treg | 10.1111/j.1365-3083.2009.02308.x |
| NKG7 | NKT cells | 10.1038/ncomms14049 |
| XCL2 | NKT cells | 10.1186/s40425-017-0215-8 |
| NCR1 | NKT cells | 10.1186/s40425-017-0215-8 |
| GNLY | NKT cells | 10.1038/ncomms14049 |
| CD79A | B-cells | 10.1038/ncomms14049 |
| LUM | Fibroblasts/myofibroblasts | 10.1016/j.jid.2018.01.016 |
| DCN | Fibroblasts/myofibroblasts | 10.1016/j.jid.2018.01.016 |
| COL1A1  VWF  ENG | Fibroblasts/myofibroblasts  Endothelium  Endothelium | 10.1016/j.celrep.2018.03.010  10.1155/2017/9759735  10.1155/2017/9759735 |
| TPSAB1 | Mast cells | 10.1186/s40425-017-0215-11 |
| PDGFRB | Pericytes | 10.1096/fj.02-0340fje |
| RGS5 | Pericytes | 10.1096/fj.02-0340fje |
